# Deep migration learning-based recognition of diseases and insect pests in Yunnan tea under complex environments

**DOI:** 10.1186/s13007-024-01219-x

**Published:** 2024-07-05

**Authors:** Zhaowen Li, Jihong Sun, Yingming Shen, Ying Yang, Xijin Wang, Xinrui Wang, Peng Tian, Ye Qian

**Affiliations:** 1 College of Big Data, Yunnan Agricultural University, Kunming, 650201 Yunnan China; 2 Yunnan Key Laboratory of Crop Production and Smart Agriculture, Yunnan Agricultural University, Kunming, 650201 Yunnan China; 3 College of Agronomy and Biotechnology, Yunnan Agricultural University, Kunming, 650201 Yunnan China; 4 International Cooperation Office, Yunnan Provincial Academy of Science and Technology, Kunming, 650201 Yunnan China; 5 College of Zoology, Yunnan Agricultural University, Kunming, 650201 Yunnan China; 6 CMCC (Shanghai) Information and Communication Technology Co, Pudong, 200120 Shanghai China

**Keywords:** Convolutional neural network, Big leaf kind of tea, Identification of diseases and pests, Transfer learning, Complex environment

## Abstract

**Background:**

The occurrence, development, and outbreak of tea diseases and pests pose a significant challenge to the quality and yield of tea, necessitating prompt identification and control measures. Given the vast array of tea diseases and pests, coupled with the intricacies of the tea planting environment, accurate and rapid diagnosis remains elusive. In addressing this issue, the present study investigates the utilization of transfer learning convolution neural networks for the identification of tea diseases and pests. Our objective is to facilitate the accurate and expeditious detection of diseases and pests affecting the Yunnan Big leaf kind of tea within its complex ecological niche.

**Results:**

Initially, we gathered 1878 image data encompassing 10 prevalent types of tea diseases and pests from complex environments within tea plantations, compiling a comprehensive dataset. Additionally, we employed data augmentation techniques to enrich the sample diversity. Leveraging the ImageNet pre-trained model, we conducted a comprehensive evaluation and identified the Xception architecture as the most effective model. Notably, the integration of an attention mechanism within the Xeption model did not yield improvements in recognition performance. Subsequently, through transfer learning and the freezing core strategy, we achieved a test accuracy rate of 98.58% and a verification accuracy rate of 98.2310%.

**Conclusions:**

These outcomes signify a significant stride towards accurate and timely detection, holding promise for enhancing the sustainability and productivity of Yunnan tea. Our findings provide a theoretical foundation and technical guidance for the development of online detection technologies for tea diseases and pests in Yunnan.

**Supplementary Information:**

The online version contains supplementary material available at 10.1186/s13007-024-01219-x.

## Introduction

China, renowned for its vast tea plantations and robust consumption, boasts a planting area of 43.956 million *mu*, playing a pivotal role in propelling agricultural growth within the country. Yunnan, a prime tea-producing region, also serves as a global hub for Pu'er tea cultivation and trade. Statistics reveal that in 2020, Yunnan's tea plantations spanned 479,800 hectares, generating an output of 466,000 tons, topping the nation in both area and production. With an average annual yield of 970.35 kg per hectare and an annual output value of 39,000 yuan per hectare [1], tea has emerged as a cornerstone industry in Yunnan, generating over 100 billion yuan in revenue. Pu'er tea, the epitome of Yunnan's tea heritage, primarily relies on big-leaf sun-dried green tea as its raw material. Consequently, the quality and yield of Pu'er tea are intimately linked to the characteristics of this specific tea variety, thereby exerting a profound influence on Yunnan's economic development and the livelihoods of farmers and related enterprises. Given that tea diseases and pests are detrimental to the growth and development of tea trees, leading to a decline in quality and yield, it is imperative to address these challenges effectively. Traditional methods of pest control, while sometimes effective, often have inherent limitations. Therefore, this research aims to delve into the strengths and weaknesses of these traditional approaches and, leveraging artificial intelligence technology, proposes an intelligent model that can circumvent their shortcomings. This model strives to achieve intelligent, rapid, and accurate identification of tea diseases and pests in the complex planting environment, paving the way for more sustainable and efficient tea production in China.

Machine learning techniques have the capability to significantly enhance model performance by exploring the intricate nonlinear relationships between prediction factors and targets [2, 3]. Consequently, they have gained widespread adoption in the realm of crop diseases and pests, encompassing diverse crops such as tobacco [4], rice [5, 6], corn [7], wheat [8], sugarcane [9], panax notoginseng [10], cucumber [11], and tomato[12]. The primary research approach involves constructing feature vectors based on characteristics like color, shape, and texture associated with the diseases. Subsequently, algorithms such as random forest [13] and support vector machine (SVM) [14] are employed for classification. Nevertheless, these traditional methods are fraught with challenges such as inconsistent feature extraction, limited adaptability, sensitivity to missing data, and overfitting issues in complex classification or regression tasks [15]. For instance, Liang Jiahui et al. [16] developed a disease spot image recognition model for wheat leaf disease, leveraging various traditional machine learning algorithms. Although the SVM model demonstrated the highest recognition accuracy of 91.31%, the traditional image recognition process involves numerous steps and methods, making it challenging to select the most suitable approach and limiting the ability to achieve automatic feature extraction. Similarly, Vimal K. Shrivastava et al. [17] introduced a rice disease classification model utilizing only color features from 619 image datasets, achieving a classification accuracy of 94.65%. However, this approach still relies on manual feature extraction, which can be both tedious and prone to human error. Kumar K Kishore et al. [18] and other researchers have also explored the application of machine learning in crop disease detection, but they have encountered similar limitations associated with traditional methods. Given these challenges, there is a pressing need to explore more advanced techniques, particularly those leveraging artificial intelligence, to overcome these limitations and enhance the accuracy and efficiency of crop disease detection and classification.

With the rapid advancement of machine learning technologies, deep learning has emerged as a focal point of research in computer vision and image recognition [19]. Among the various deep learning techniques, the Convolutional Neural Network (CNN) model has garnered significant attention. This multi-layer neural network, developed in recent years, has found widespread application in diverse fields such as image processing and natural language processing (NLP). Its forward neural network architecture and deep learning capabilities enable it to achieve optimized performance and mitigate overfitting through techniques such as weight sharing, local connections, and pooling. Furthermore, CNN's ability to learn characteristics across different fields, scenes, and scales facilitates end-to-end detection, making it a valuable tool in crop pest identification, where its application is gradually becoming more widespread [20–24]. For instance, Lin Sen et al. [25] proposed a fine-grained pest identification method utilizing a Graph Pyramid Attention Convolutional Neural Network (GPA-Net). Comparative experiments conducted on cassava leaves, AI Challenger, and IP102 pest datasets demonstrate that the GPA-Net outperforms existing models, achieving accuracy rates of 99.0%, 97.0%, and 56.9%, respectively. Similarly, Lin Yuke et al. [26] employed neural networks to segment disease spots in citrus images, achieving accurate recognition results through feature matching. Their comparative experimental results reveal that this method improves the recognition rate by approximately 11.9% compared to traditional recognition methods, exhibiting superior performance. Nigam Sapna et al. [27] introduced a wheat disease identification model based on the EfficientNet architecture, designed to automatically detect major wheat rust. The fine-tuned EfficientNet B4 model achieved a remarkable test accuracy of 99.35%. Moreover, Guangsheng Liu et al. [28] proposed a lightweight model known as the Selective Kernel Mobile Network (SK MobileNet). This model achieves an accuracy rate of 99.28% on public datasets while being sufficiently lightweight to significantly reduce computing costs when deployed on servers. These advancements demonstrate the potential of deep learning techniques, particularly CNNs, in enhancing the accuracy and efficiency of crop diseases and pests detection. C.K. Sunil et al. [29] have sorted out the challenges and opportunities of plant disease detection and proposed an integrated deep learning based plant disease diagnosis method based on a combination of AlexNet, ResNet50 and VGG16 deep learning models, the maximum detection accuracy of the proposed method for binary dataset is 100% and for multi-class dataset is 99.53%.

In the realm of tea disease and pest recognition, Santhana Krishnan Jayapal et al. [30] introduced an image retrieval model specifically tailored for tea disease recognition. This model, leveraging depth hash and an integrated self-encoder, exhibits superior MAP (Mean Average Precision) scores compared to state-of-the-art methods. On the other hand, Lanjewar Madhusudan G [31] and his team proposed the development and implementation of a real-time disease prediction system, employing convolutional neural networks (CNN) on a Platform as a Service (PaaS) cloud. Li Zimao and colleagues [32] introduced an SE DenseNet FL approach for tea disease recognition, grounded in transfer learning. Notably, this method achieved a remarkable recognition accuracy rate of 92.66% even under challenging conditions of limited and unevenly distributed samples. Mukhopadhyay Somnath et al. [33] put forth an image clustering method leveraging a non-dominated sorting genetic algorithm (NSGA-II) for detecting tea disease areas. Experimental results demonstrated the algorithm's ability to detect persistent disease types in tea, achieving an average accuracy of 83%. Furthermore, Gensheng Hu and his team [34] proposed a tea disease recognition method based on an improved deep convolutional neural network (CNN), achieving an average recognition accuracy of 92.5%. Lu Bing et al. [35] introduced a spectral extraction method focused on the region of interest within the speckle region. This approach enabled the neural network structure-based ELM model to attain a classification accuracy of 95.77%. These advancements not only demonstrate the significant progress made in the field of tea disease and pest recognition but also highlight the potential of deep learning techniques in enhancing the accuracy and efficiency of such recognition systems.

Nevertheless, there are some limitations in the application of convolutional neural networks (CNN) in the field of crop disease and pest recognition. While a large amount of training data is essential to train an accurate and efficient recognition model, the collection of crop disease and pest image data is difficult, time-consuming and labor-intensive. Additionally, the demand for training data and computational resources during the development process is quite large, which may be difficult to fulfill in some application scenarios. Therefore, improving the efficiency of CNN development has become an urgent problem currently. Based on the previous research results and the current development of China's tea industry, this study aims to combine deep learning algorithms to develop an automatic classification model of disease and pest digital images for small samples of Yunnan Big leaf kind of tea in complex environments, and at the same time, explore ways to improve the development efficiency of CNNs in order to expand the feasibility of their application in small agricultural environments.

### Experimental materials and methods

During the tea planting process, an extensive collection of over 1800 original field images of tea leaves was assembled. These images were subsequently categorized into 10 distinct disease and pest groups, thus establishing a comprehensive dataset specific to Yunnan Big leaf kind of tea. Given the limited quantity and unbalanced distribution of the data, we employed data augmentation techniques to broaden the dataset's scope. Prior to training, the model was pre-trained on the ImageNet dataset, and the shared parameters obtained from this pre-training were then transferred to our tea disease and pest identification model for further fine-tuning and optimization. To assess the performance of various classification approaches, we explored and compared six high-performing image classifiers: Xeption [36], VGG-16 [37], ResNet-50 [38], Inception V3 [39], MobileNetV2 [40], and DenseNet201 [41] within the context of transfer learning. Additionally, we analyzed the impact of diverse fine-tuning strategies, varying learning rates, and various attention mechanisms on the model's classification accuracy. After a thorough evaluation, the model exhibiting the most optimal performance was chosen to develop a disease and pest classification model tailored for the limited samples of Yunnan Big leaf kind of tea in complex environments. This model was further enhanced through the integration of transfer learning techniques and an embedded attention mechanism. Finally, the effectiveness of the optimized model was rigorously verified.

### Test data collection

**Fig. 1:**
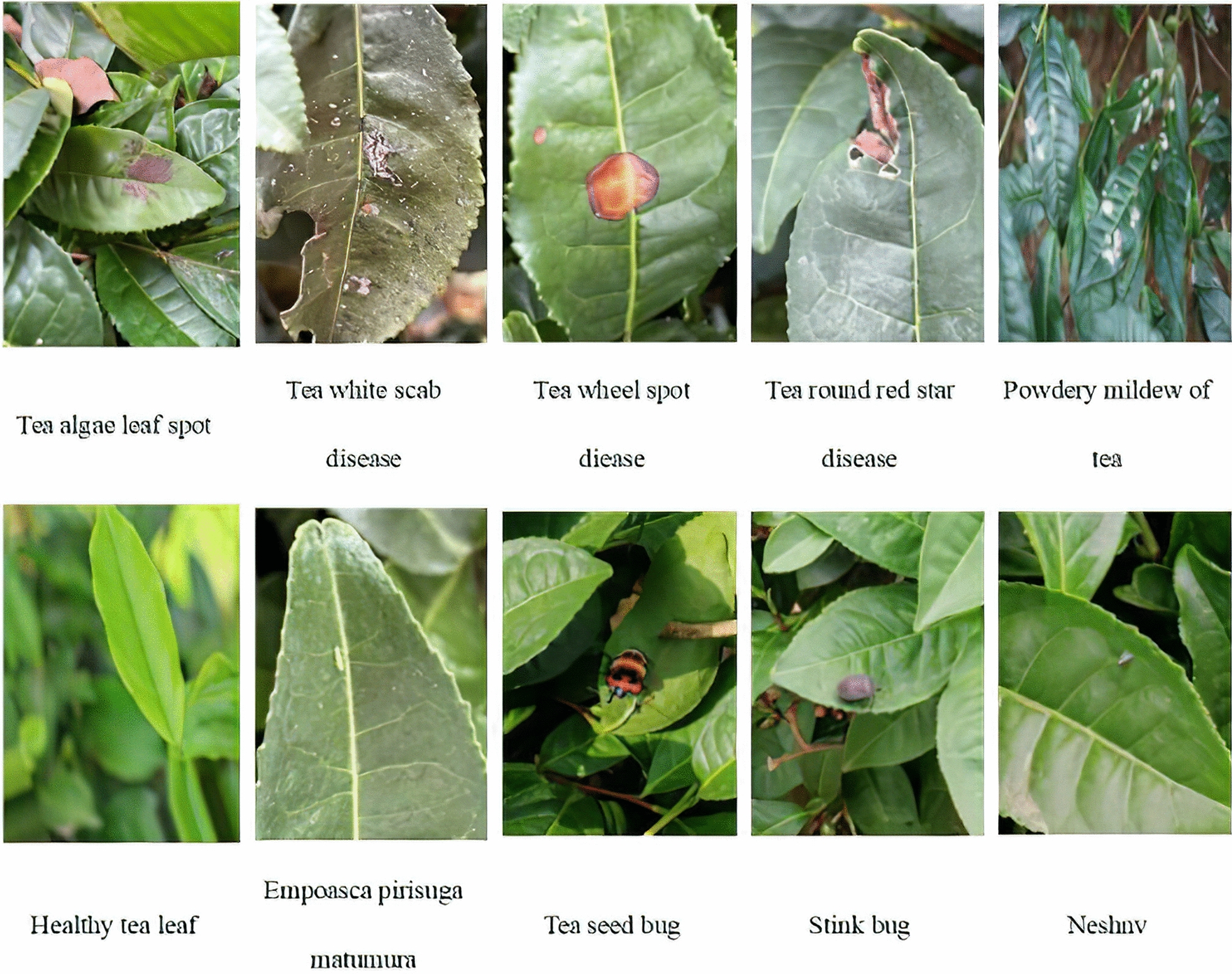
Samples of diseases and pests in large-leaf sun-dried green tea with large leaves

Gathering a comprehensive tea disease dataset in a natural environment serves as the foundation for accurate disease and pest recognition in complex settings. On June 2, 2022, the images of tea diseases used in this experiment were meticulously captured from the tea garden planting base of Longrun Pu'er Tea College of Yunnan Agricultural University. To assess the precision of capturing images in the natural environment using portable devices, a mobile phone (specifically, an Iphone13 with 12 megapixels and a Huawei P50pro with 50 megapixels) was employed as the shooting equipment. The camera was positioned 10 ~ 15 cm away from diseased tea leaves and 20 ~ 35 cm away from insect pests. Varied shooting angles, including front, overhead, and side shots, were utilized to capture the diseases and pests. The imaging background naturally included interfering elements such as tea branches, soil, and other insects, ensuring the authenticity of the disease data, as illustrated in Fig. 1. In total, 97 photos of tea algal leaf spot, 322 photos of tea white spot, 75 photos of tea wheel spot diease, 157 photos of tea round red spot disease were collected. Additionally, 101 photos of empoasca pirisuga matumura, 339 photos of tea seed bug, 42 photos of stink bug, and 93 photos of neshnv were captured to document the insect pests. Then, 385 photos of powdery mildew of tea and 261 photos of healthy tea leaf were crawled from the network to increase data diversity. This comprehensive dataset provides a robust foundation for further analysis and the development of accurate recognition models in the realm of artificial intelligence.

### Test data enhancement and data set production

**Fig. 2:**
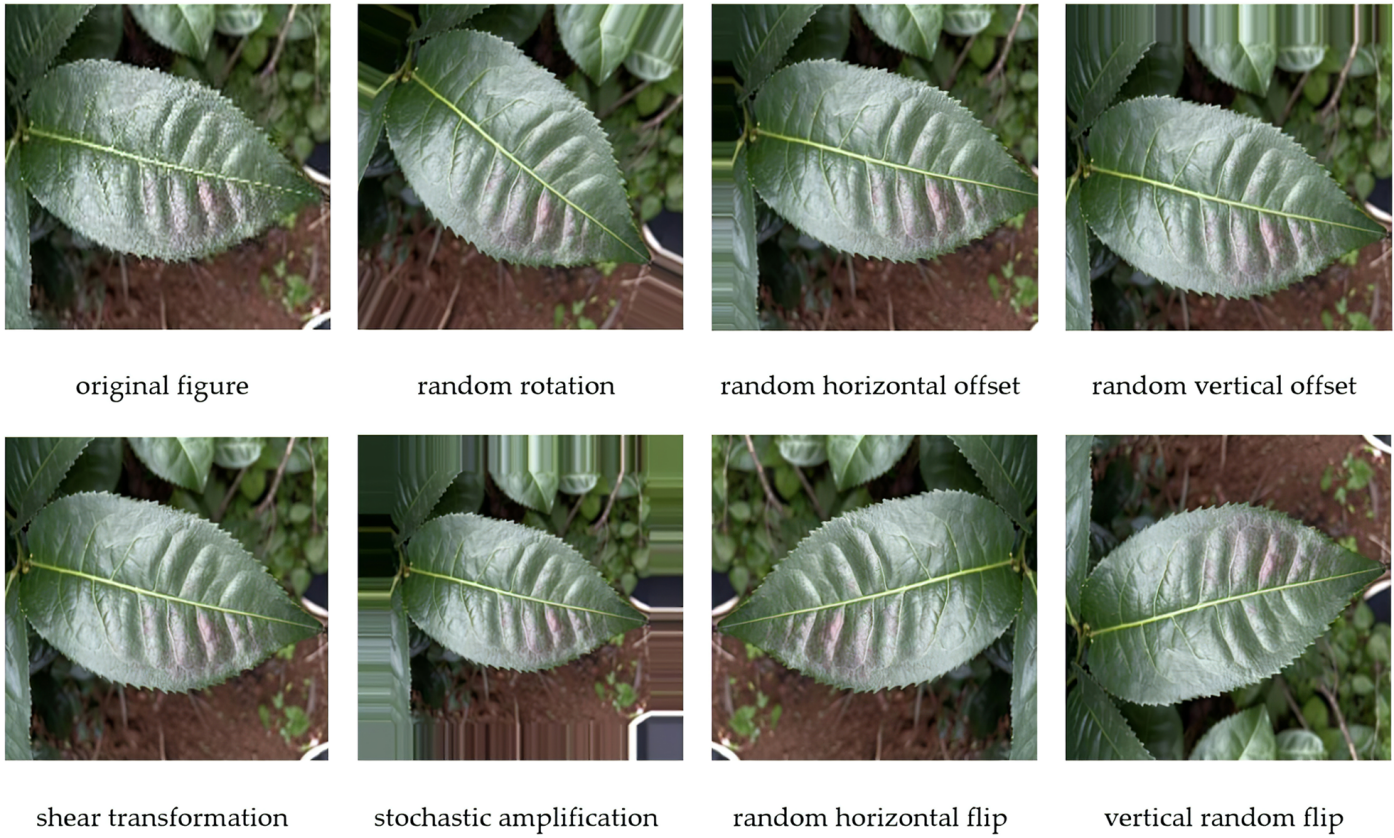
Data enhancement of sample image

Convolutional neural networks require an adequate number of samples to learn image features effectively, thereby enhancing the recognition accuracy of the target images. When the sample size is insufficient, it can significantly impact the model's training outcome, potentially leading to issues such as overfitting [42] and diminished generalization capabilities, ultimately reducing the model's practical utility. To enhance the generalization capacity of our network, we leveraged the ImageDataGenerator tool from the Keras framework in conjunction with Python. This approach allowed us to compress the image resolution to 640 × 640 pixels while simultaneously expanding the sample set through various data augmentation techniques. These techniques included random rotations of up to 30°, random horizontal and vertical shifts with an amplitude of 0.1, shear transformations with an intensity of 0.2, random zooming with an intensity of 0.2, and random horizontal and vertical flips. As depicted in Fig. 2, the images were labeled and categorized accordingly. The labels included TALS (tea algal spot disease), TWSD (tea white spot disease), TWS (tea wheel spot disease), TRRSD (tea round red star disease), PM (powdery mildew of tea), HL (healthy tea), EPM (empoasca pirisuga matumura), SB (stink bug), N (neshnv), and TSB (tea seed bug). To construct a comprehensive database of tea diseases and pests, the labeled images were imported into a computer in jpeg format. This database was then used to train the model on various diseases and pests affecting the Yunnan Big leaf kind of tea. After applying the image augmentation techniques, the resulting sample set consisted of 5650 images. These images were subsequently divided into a training set (4520 images), a test set (565 images), and a validation set (565 images), using an 8:1:1 ratio. The distribution of these data is summarized in Table 1.

**Table 1:** Data distribution

Classes	Original data set	Enhanced data sets	Training set	Test set	Validation set
TALS	97	300	240	30	30
TWSD	322	900	720	90	90
TWS	75	250	200	25	25
TRRSD	157	450	360	45	45
HL	261	800	640	80	80
PM	385	1200	960	120	120
EPM	101	300	240	30	30
SB	42	150	120	15	15
N	99	300	240	30	30
TSB	339	1000	800	100	100
Total	1878	5650	4520	565	565

### Sample normalization

Firstly, the image is resized to 640 × 640 pixels and subsequently converted to the float32 datatype. This step is crucial as the original pixel values, ranging from 0 to 255, are excessively large for efficient computer processing. To normalize the image, we divide each pixel value by 255, resulting in a normalized range of 0 to 1, which is more suitable for computational purposes. Additionally, the dataset is thoroughly shuffled to ensure randomness and prevent any biases that may arise from the order of the samples. Furthermore, the employment of a dynamic learning rate scheduling strategy effectively mitigates the potential learning imbalance caused by an over-concentration of specific features. This approach ensures the accuracy of the learning direction, preventing any deviations and promoting a more robust and generalized model.

### Transfer learning

Migration learning, a subset of transfer learning, segregates data into two categories: source data and target data. Source data typically comprises large samples unrelated to the specific research task, whereas target data comprises smaller samples that are directly pertinent to the task. The essence of transfer learning lies in leveraging knowledge derived from source data and existing models to facilitate knowledge sharing. This approach not only maximizes the utilization of source data but also enhances the performance of models on target data [43]. In the context of this study, addressing the challenges posed by the limited number of samples and uneven distribution within the tea disease and insect pest dataset, we adopt a two-stage training process. Initially, the model is pre-trained on the ImageNet dataset [44], with the pre-training weight parameters subsequently saved. Subsequently, these parameters are transferred to the tea disease and insect pest dataset for further fine-tuning. Among the six convolutional neural network classification models considered, the one exhibiting the best performance is selected. To further optimize the model's performance, we employ a strategy known as "low-level freezing, high-level training." This approach involves freezing the parameters and structure of the lower layers of the model, while utilizing the pre-processed tea data for training the higher layers. This allows the model to learn the deeper characteristics associated with different diseases and pests, thereby enhancing its discriminative ability and overall performance.

### Attention mechanism

The attention mechanism, which mimics the workings of biological vision, has garnered widespread attention in image recognition tasks due to its computational efficiency and profound understanding of image content [45]. Central to its operation is the ability to weight the output of neurons not solely based on the output of the preceding layer's neurons, but also according to distinct sections of the input data. This approach enables the model to prioritize key information within the input sequence, thereby enhancing both accuracy and efficiency. In the realm of image processing, three primary attention mechanisms stand out: SENet, ECANet, and CBAM. The SE attention mechanism introduces an attention mechanism in the channel dimension. By leveraging automatic learning, a supplementary neural network is employed to assess the significance of each channel in the feature map. Subsequently, this significance is utilized to assign weight values to individual features, allowing the neural network to concentrate on particular feature channels [46]. The ECA attention mechanism module incorporates a 1 × 1 convolution layer directly after the global average pooling layer, eliminating the need for a fully connected layer. This design choice circumvents dimension reduction and effectively captures cross-channel interactions. Notably, ECANet achieves impressive results with minimal parameters [47]. CBAM operates by initially passing the input feature map through the channel attention mechanism. The resulting channel weights are then multiplied with the input feature map, and the combined output is forwarded to the spatial attention mechanism. Here, the normalized spatial weights are multiplied with the input feature map of the spatial attention mechanism to yield the final weighted feature map [48]. In this study, we sought to assess the performance of various attention mechanisms on a challenging tea dataset with limited samples and a complex environmental backdrop. To achieve this, we integrated the three attention mechanisms into the best-performing model and evaluated their effectiveness.

### Technical lines of research

**Fig. 3:**
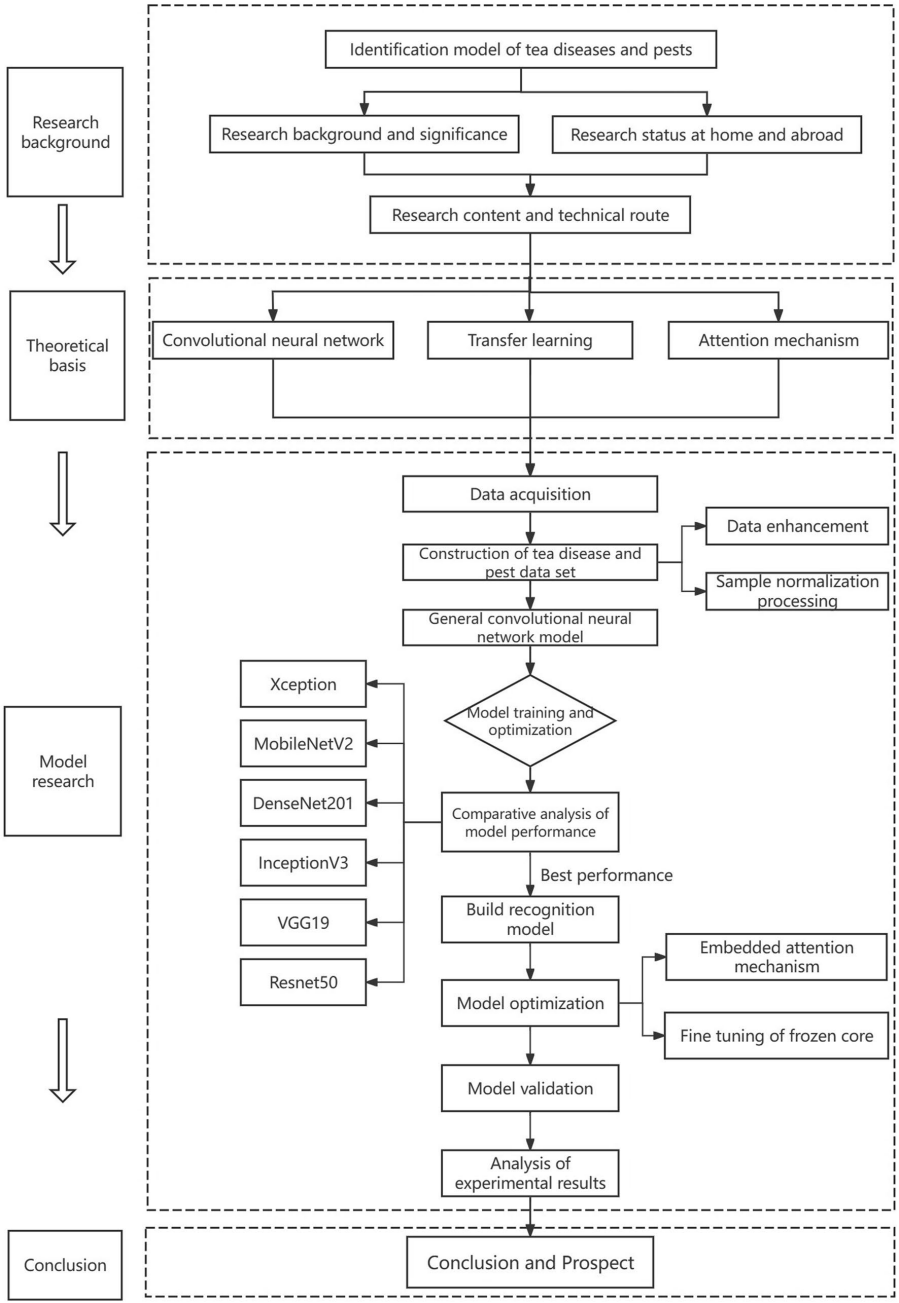
Technology roadmap

The technical route of this article is shown in Fig. 3.

1. Research background analysis and grasp the relevant theoretical basis.
  First of all, the research content was identified as the identification of diseases and pests of Yunnan big leaf tea plants. Then, the relevant knowledge of tea diseases and pests and in-depth learning related books were consulted to master the characteristics of common diseases and pests of tea. Through literature analysis, the research status in the field of tea diseases and pests identification was analyzed, and the in-depth learning methods and frameworks suitable for this task were studied.
2. Data acquisition and data pre-processing.
  Images of diseased leaves and pests of large leaf tea plants in the tea garden planting base of Yunnan Agricultural University were collected. After image acquisition, the ImageDataGenerator generator in the keras framework was used to preprocess the images, and the sample set was expanded through image random rotation, random offset, shear transformation and other data enhancement methods to build a large leaf tea plant disease and pest data set.
3. Model construction and optimization.
  Combined with the task and data set feature analysis in this paper, we comprehensively compared the performance of Xception, VGG-16, ResNet-50, Inception V3, MobileNetV2 and DenseNet20 in migration learning, selected the best performance model to build a disease and pest classification model for the leaves of small sample Yunnan large leaf tea plants in complex environments, and then optimized the model through migration learning and embedded attention mechanism, and verified the effectiveness of the model.
4. Model validation and experimental results analysis.
  For the optimized model, the validation set is used to predict, and the confusion matrix is drawn to objectively and comprehensively evaluate its recognition performance and classification results, further verifying the effectiveness of the model in this paper.

### Model design

#### Training

**Fig. 4:**
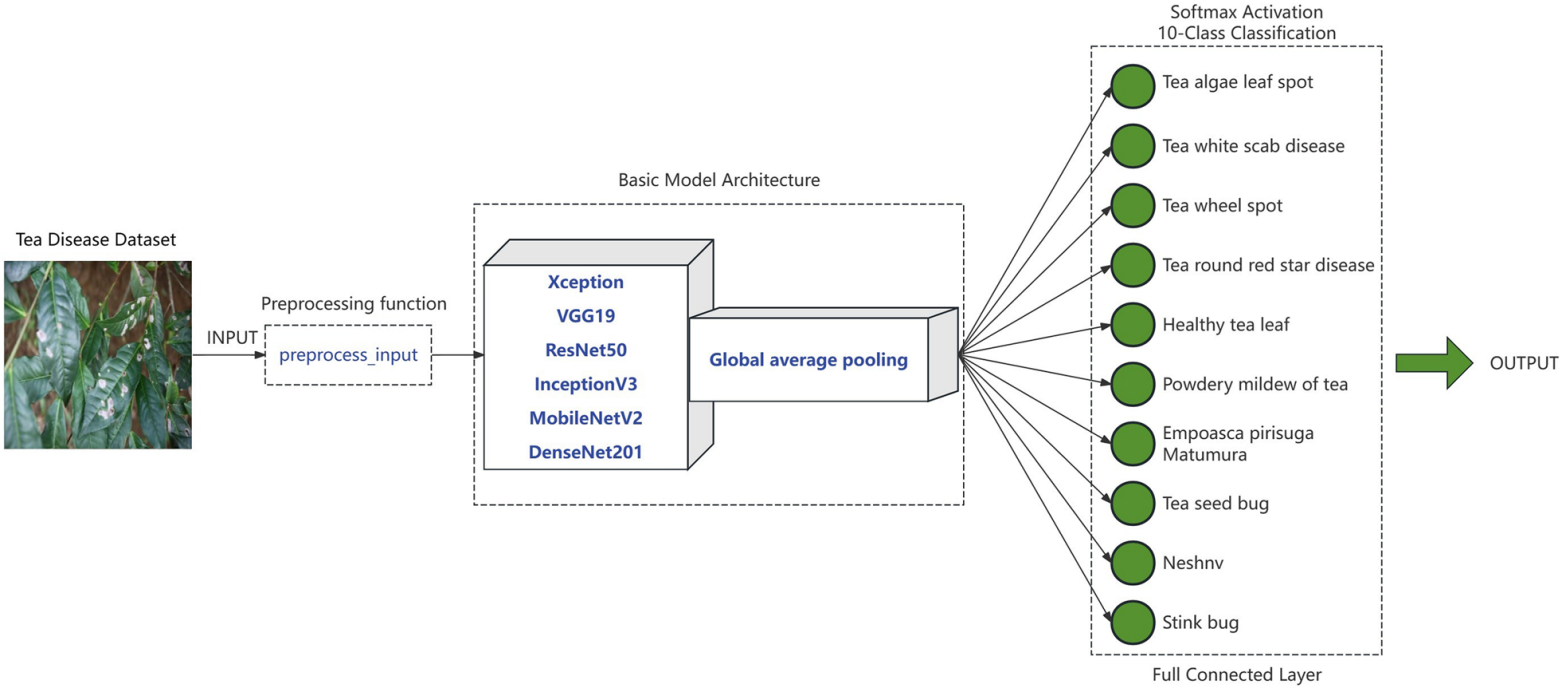
Convolutional neural network model architecture

Utilizing the transfer learning approach, we fine-tuned the weights of a pre-trained basic CNN architecture, originally trained on the ImageNet dataset, for application on the tea image dataset. We conducted a comparative analysis of various common CNN architectures, evaluating their performance across different model parameters and data augmentation techniques. Based on this analysis, we designed a unified model architecture as depicted in Fig. 4. Initially, the pre-processed and standardized dataset is fed into the model, excluding the top layer, enabling feature extraction through the convolutional layers. Subsequently, global average pooling is employed to achieve feature dimensionality reduction. Finally, a softmax layer is utilized for classifying tea diseases and pests. This approach ensures that each feature map is directly associated with the classification probability, eliminating the need for a fully connected layer and thus mitigating the generation of additional training parameters. This reduction in parameters minimizes the risk of overfitting [49].

The computer operating system used for model training and testing is Windows11 64 bit system with 16 GB memory and AMD Ryzen 7 7735HCPU@3.20 GHz Processor, NVIDIA GeForce RTX4060 graphics card. The IDE is PyCharm2022, with Python 3.8.16, tensorflow-gpu2.6.0, and keras2.6.0 configured.

#### Network super parameter setting

The terminal output layer of the model comprises 10 neurons, corresponding to 10 distinct categories, and employs the softmax function as its activation mechanism. For both model training and testing, a GPU processor is utilized, while the Adam optimizer is chosen as the model optimizer. Additionally, a batch training approach is adopted, with each batch containing 64 samples. Overall, 100 iterations are performed to ensure comprehensive model training. In the fine-tuning phase of transfer learning and the integration of the embedded attention mechanism, a learning rate of 0.001 is employed. Furthermore, a dynamic learning rate scheduling strategy is implemented in the subsequent optimization stage, aiming to expedite model convergence and enhance overall performance.

### Evaluation index

The final model was tested and validated using 565 pictures from the validation set, and evaluated using quantitative indicators derived from the confusion matrix. These indicators include accuracy rate, accuracy rate, recall rate and F-1 score. Accuracy is a common performance indicator in classification problems, which measures the overall performance of the model on all samples:

1 $$Accuracy=\frac{TP+TN}{TP+FP+TN+FN}$$

Accuracy is used to evaluate the accuracy of prediction by comparing the number of correctly predicted category images with the total number of the category images predicted by the model:

2 $$Precision=\frac{TP}{TP+FP}$$

The recall rate mainly measures the proportion of predicted positive samples among the actual positive samples:

3 $$Recall=\frac{TP}{TP+FN}$$

The F1 score is the harmonic average of the accuracy rate and recall rate, which takes into account the proportion of the real samples in the model prediction results and the proportion of the predicted positive samples in the actual positive samples:

4 $$F1-Score=2\times \frac{Precision\times Recall}{Precision+Recall}$$

TP (True Positives) is the number of samples correctly predicted by the model as positive examples; FP (False Positives) is the number of samples incorrectly predicted by the model as positive examples; TN (True Negatives) is the number of samples correctly predicted by the model as negative cases; FN (False negatives) is the number of samples whose model incorrectly predicts negative cases.

FLOPs stands for floating point operations (s-table complex), i.e., the number of floating point operations, is a measure of the total number of operations that a model must perform in order to perform classification, and can be used to measure the complexity of the model, with higher values representing a higher number of floating point operations required to complete a forward propagation, and a lower efficiency of the model.

### Experimental results and analysis

#### Performance improvement analysis of models before and after data enhancement

**Fig. 5:**
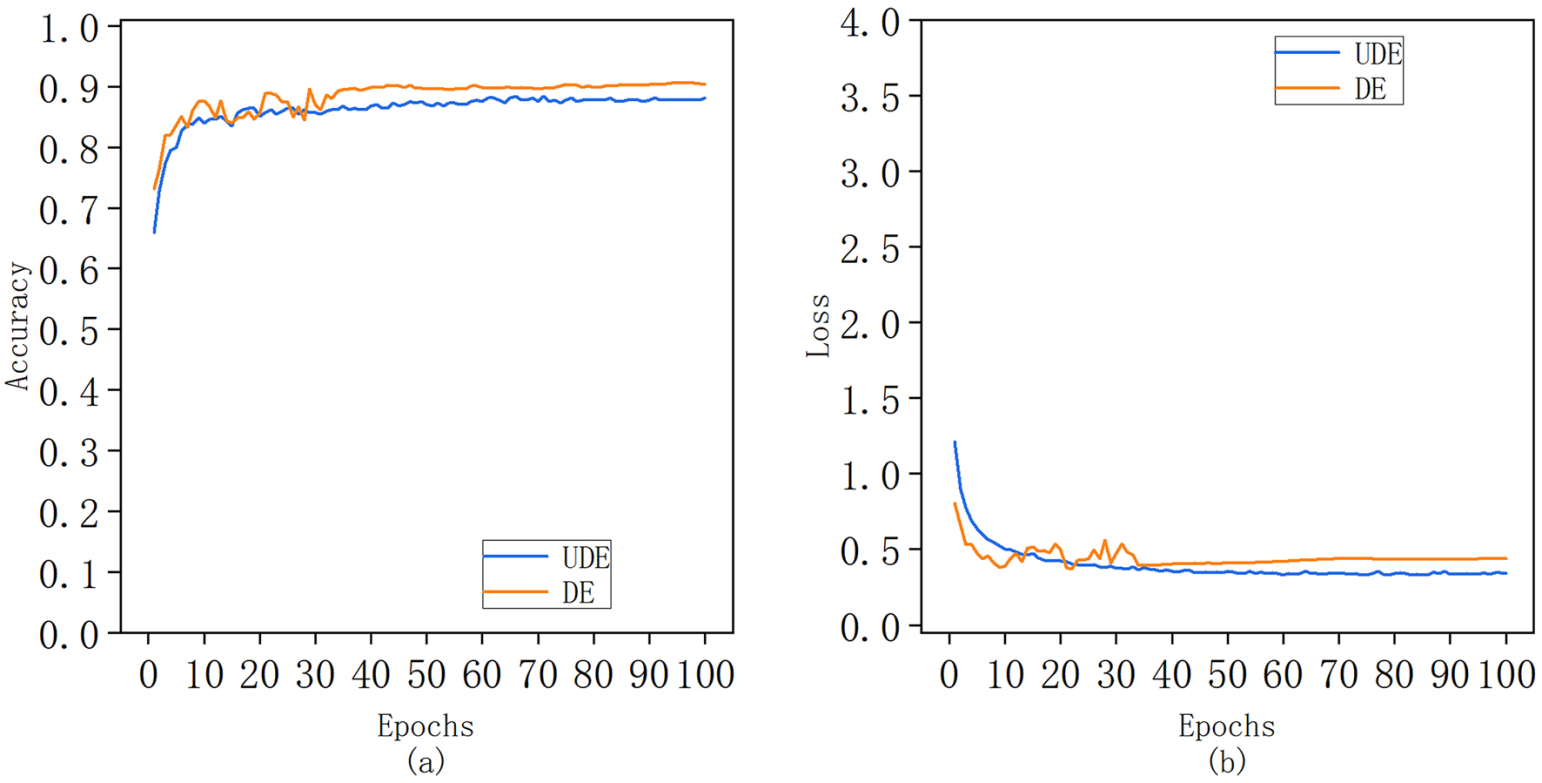
Curve Chart of Accuracy and Loss Value of Xception Model before and after Data Set Enhancement (UDE stands for not using data enhancement, DE stands for using data enhancement)

Figure 5 compares the accuracy and loss values of the Xception model before and after dataset enhancement. From the graph, the gap between the two is not big. Both start to converge at 35 iterations, and the UDE accuracy is only about 2% lower than DE. It can be seen from Table 2 that the model efficiency of UDE is far lower than that of DE. The time of each step of UDE is more than 8 times higher than that of DE, and the time of each round is 48 s longer than that of DE.

**Table 2:** Performance comparison of Xoption model before and after dataset enhancement

Model	Average precision (%)	Average loss	Time of each epoch (s)	Time of each epoch (s)
DE	90.04	0.0849	3000	27
UDE	87.84	0.0440	372	75

To sum up, data enhancement generates new training samples by applying various random transformations (such as rotation, scaling, flipping, cropping, etc.), so that the model will be exposed to more different variants during training, significantly increasing the generalization ability of the model while saving data collection costs.

#### Model performance comparison

**Fig. 6:**
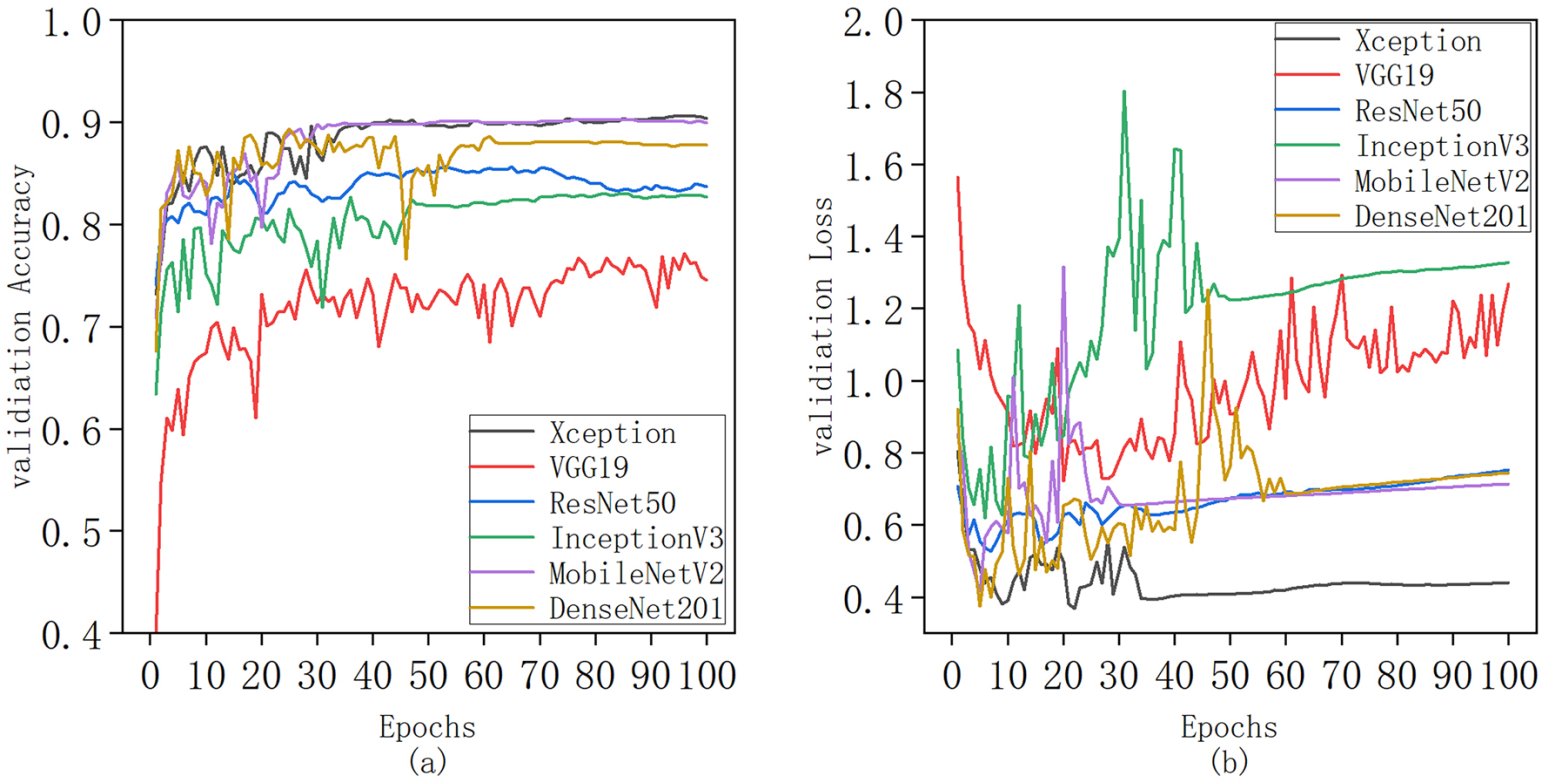
Comparison of accuracy and loss function values: (**a**) Curve of change in accuracy for the test set; (**b**) Curve of change in loss function values for the test set

Under identical parameter settings and experimental conditions, Fig. 6 presents the training outcomes of various models—Xeption, VGG19, ResNet50, Inception V3, MobileNetV2, and DenseNet201—on the designated test set. As evident in Fig. 6a, the accuracy of each model demonstrates a gradual increase with the growing number of iterations. Notably, after 50 epochs of training, the accuracy of VGG19 experienced significant fluctuations, whereas the accuracy curves of the remaining models converged and stabilized. The peak recognition accuracies achieved by these six models were 90.62%, 90.2%, 87.78%, 83.38%, 82.81%, and 77.13%, respectively. Figure 6b illustrates the loss trends during training. Notably, the VGG19 model continued to exhibit significant fluctuations throughout the training process. In contrast, the loss values of the other models exhibited a steep decline and fluctuations during the initial 60 epochs, subsequently diminishing gradually, and ultimately attaining exceptionally low values. Among them, the Xeption model exhibited smoother loss curves and lower loss values compared to the other five models.

Table 3 comprehensively outlines the comparative performance of six transfer learning architectures, encompassing evaluation metrics such as average precision, average loss, step time, round time, total parameters, trainable parameter quantities, and untrainable parameter quantities. Among these models, MobileNetV2 emerges as the top performer with an average precision of 90.05%, closely followed by Xeption, DenseNet201, and ResNet50, each achieving 90.04%. When considering the loss function value, the Xeption model distinguishes itself with the lowest and most stable loss of 0.4304, indicating its superiority in terms of model convergence and prediction accuracy.

**Table 3:** Comparison of model performance (average precision and average loss refer to the average after model convergence, both after 50 epochs)

Model	Average precision (%)	Average loss	Time of each step (ms)	Time of each epoch (s)	Depth	Total Parameters	Trainable Parameters	Non-trainable Parameters	FLOPs(G)
Xception	90.04	0.4304	372	26	81	20,881,970	20,490	20,861,480	16.7
VGG19	74.35	1.0884	225	16	19	20,029,514	5130	20,024,384	39
ResNet50	84.48	0.7094	180	13	107	23,608,202	20,490	23,578,712	7.7
InceptionV3	82.49	1.2836	220	15	189	21,823,274	20,490	21,802,784	11.4
MobileNetV2	90.05	0.6932	100	7	105	2,270,794	12,810	2,257,984	0.6
DenseNet201	87.72	0.7255	270	20	402	18,341,194	19,210	18,321,984	8.6

In summary, the Xeption model exhibits outstanding performance in terms of both accuracy and loss value. This is attributed to its lightweight design, shallow network structure, and balanced parameterization. Given the limited quantity and uneven distribution of samples in the tea disease and pest dataset, the Xeption model demonstrates exceptional performance, making it the preferred choice for addressing this specific task.

#### Analysis on the improvement of recognition performance of tea diseases and pests after embedding the attention mechanism into the Xoption based model

The attention mechanism effectively enhances the activation weight of tea disease and pest-affected areas, mitigating the interference from background information. This allows the network to precisely identify the disease and pest regions that have the most significant impact on recognition outcomes. Consequently, prior to integrating SeNet, ECANet, and CBAM into the ultimate global average pooling layer of the Xception model, Fig. 5 illustrates the training outcomes achieved by employing these three methodologies.

After selecting the basic model as the Xeption model, we first thought of introducing the attention mechanism to improve the accuracy of tea disease and pest identification models. Because the placement of the attention mechanism block on the backbone will result in the inability to use the network's pre training weight, this study places the attention mechanism block before the final global average pooling layer, as shown in Table 4.

**Table 4:** Attention Xeption Model

Module	Input	Operator	Repeat	Output channels
Entry flow	299 × 299 × 3			32
	149 × 149 × 32	Conv2D		64
	147 × 147 × 64	Conv2D		128
	147 × 147 × 128	SeparableConv2		128
	74 × 74 × 128	Conv2D		256
	74 × 74 × 256	SeparableConv2	2	256
	37 × 37 × 256	Conv2D		728
	37 × 37 × 728	SeparableConv2	2	728
	19 × 19 × 728	Conv2D		728
Middle flow	19 × 19 × 728	SeparableConv2	25	1024
Exit flow	19 × 19 × 1024	SeparableConv2		1024
	10 × 10 × 1024	Conv2D		1536
	10 × 10 × 1536	SeparableConv2		2048
	10 × 10 × 2048	SeparableConv2		2048
	10 × 10 × 2048	SE/ECA/CBAM block		2048
	10 × 10 × 2048	GlobalAveragePooling2		2048
	2048	Dense		10

**Fig. 7:**
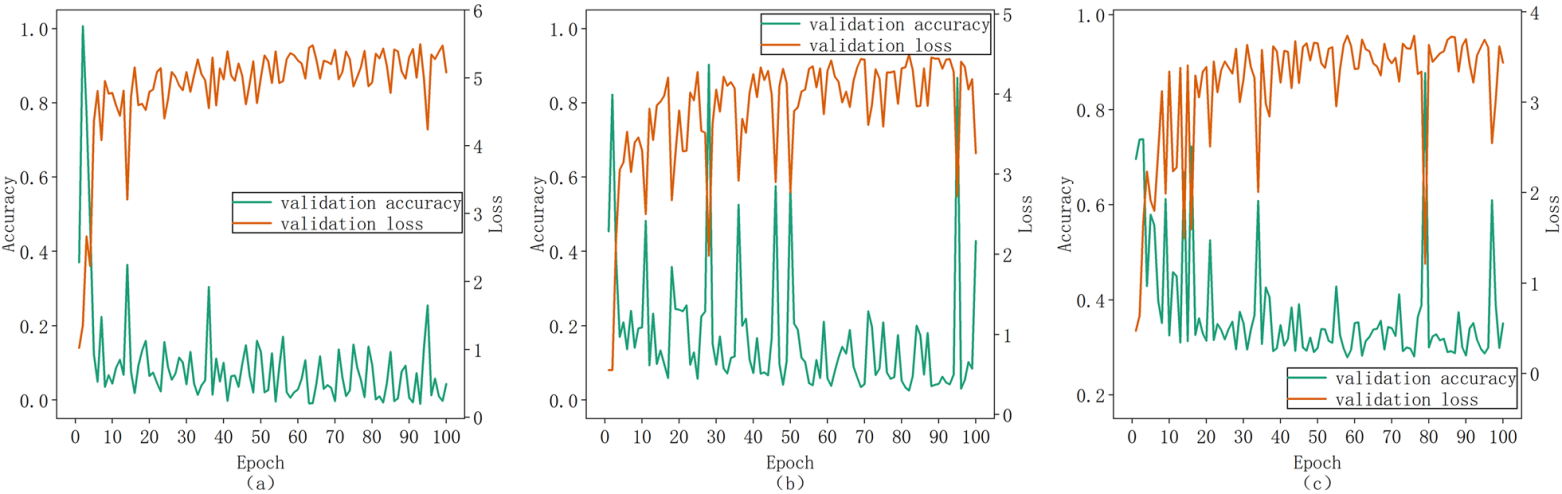
Comparison of accuracy and loss under different attention mechanism approaches: (**a**) Xception_SeNet; (**b**) Xception_ECANet (**c**) Xception_CBAM

As evident from Fig. 7, the introduction of the attention mechanism has indeed enhanced the recognition accuracy of each model: Xception_SeNet achieved the highest accuracy rate of 95.8%, while Xception_ECA reached 91.81%, and NetXception_CBAM attained 91.43%. However, it is noteworthy that the models exhibited significant fluctuations and were unable to converge stably, resulting in a severe overfitting phenomenon. Based on our analysis of the dataset, we speculate that the following issues may have contributed to this: (1) Prior to incorporating the attention mechanism, the model was already in a state of overfitting. The addition of further parameters exacerbated this issue, leading to a decline in performance. (2) Excessively deep layers and numerous channels are prone to causing overfitting. (3) Given the small size of the feature map, improper operations during convolution may introduce a significant amount of non-pixel information. (4) The proximity of the attention mechanism to the classification layer renders its effect highly sensitive to classification outcomes, potentially influencing decision-making at the classification level.

#### Result analysis of "low level freezing and high level training"

**Fig. 8:**
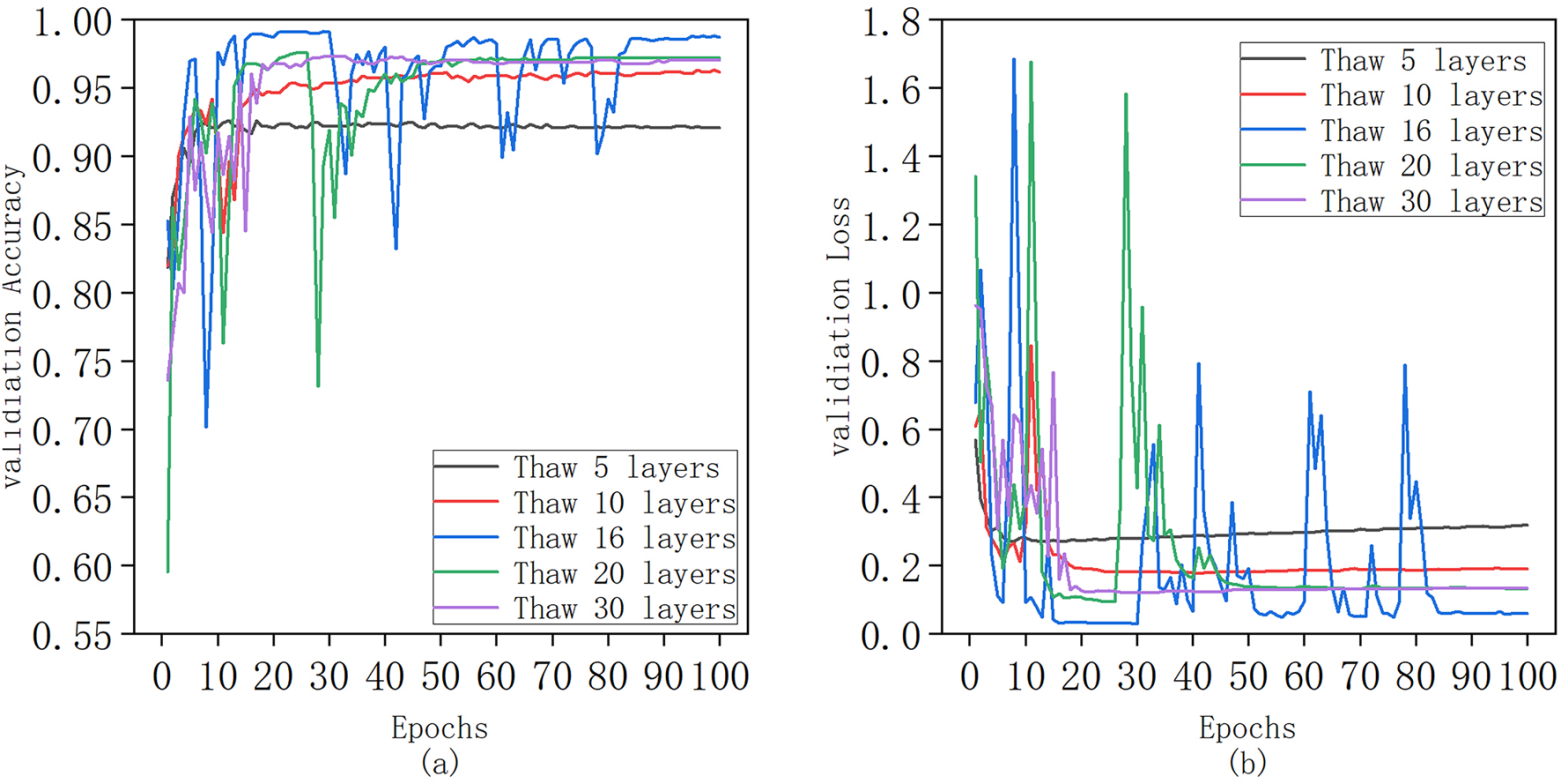
Comparison of model accuracy and loss function values under different fine-tuning strategies: (**a**) Curve of change in accuracy of test set; (**b**) Curve of change in loss function value of test set

After comparing the Xeption model's performance with the integration of the attention mechanism, we adopted a different approach to further optimize the experiment: the fine-tuning strategy of "freezing lower levels, training higher levels." Initially, we unfroze all the convolution layers in the Xeption model. Subsequently, we focused on fine-tuning specific layers while freezing the remaining ones. More specifically, we trained the top 5, 10, 16, 20, and 30 layers of the unfrozen model, and the results are presented in Fig. 8. The experimental outcomes reveal that the fine-tuning strategy significantly enhances the model's precision. The accuracy rates, from highest to lowest, are: 98.71% for the unfrozen 16 layers, 97.16% for the unfrozen 20 layers, 97.02% for the unfrozen 30 layers, 96.16% for the unfrozen 10 layers, and 92.05% for the unfrozen 5 layers. This improvement can be attributed to the strong correlation between the specific features extracted from the deeper layers of the neural network and the target task. By reinitializing and retraining these network parameters, we can better adapt them to new tasks.

**Fig. 9:**
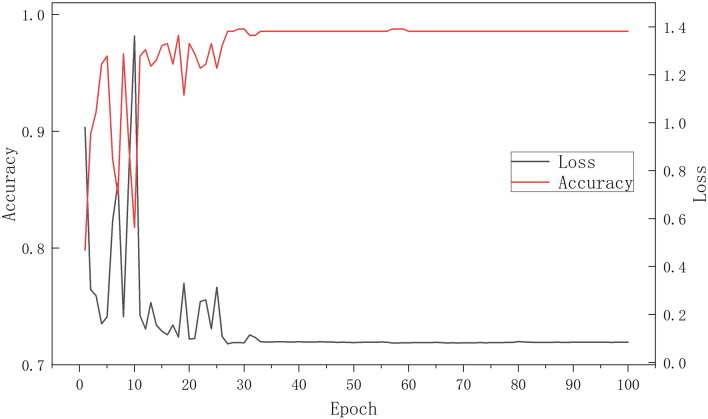
Accuracy and loss function values of the model with 16 layers unfrozen and a learning rate of 0.001 to 0.0001 dynamically descending selected

However, during the thawing process of the 16 layers, we observed significant fluctuations in the training results. We hypothesized that the use of a fixed learning rate might have led to the later parameters oscillating around the optimal solution. Initially, a larger learning rate is essential to quickly approach the optimal solution. Subsequently, a reduced learning rate is required to fine-tune and obtain a more precise optimal solution. Therefore, we subsequently employed a callback function to dynamically adjust the learning rate during training. We set the detection criterion as the accuracy on the test set, with an initial learning rate of 0.001 and a decay factor of 0.5. If the detection value fails to improve after 8 epochs, the learning rate is halved until it reaches 0.0001. The experimental results, presented in Fig. 9, demonstrate that the model achieved a maximum accuracy of 98.58% with a minimum loss value of 0.0849. Although the model exhibited some oscillations before 15 epochs, it converged and stabilized after 20 epochs, indicating the effectiveness of our dynamic learning rate scheduling approach.

#### Verification and analysis of disease and pest identification model of Big leaf kind of tea

**Fig. 10:**
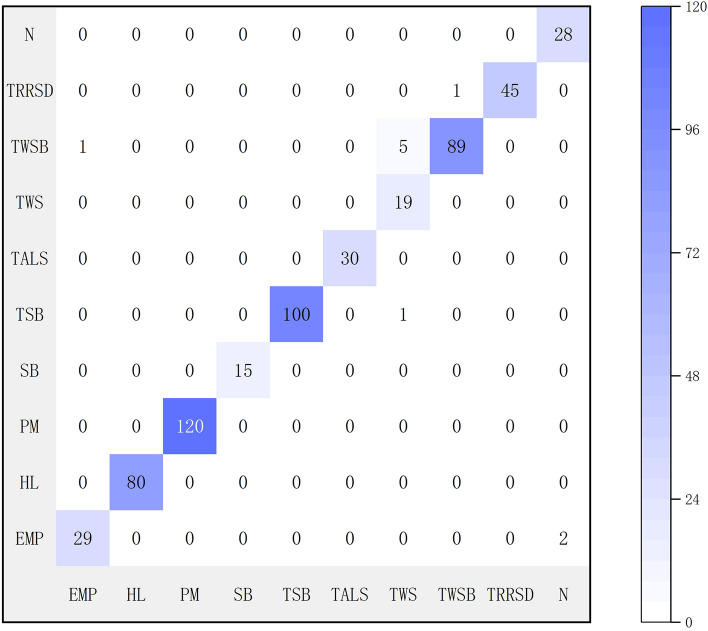
Confusion matrix for Xception model with 16 layers of fine-tuning

To assess the recognition performance and classification outcomes objectively and comprehensively using the Xception model, this study evaluated the model using a validation set of 565 images. A confusion matrix (illustrated in Fig. 10) was constructed based on the classification results, providing detailed insights into the prediction outcomes of the validation dataset (Table 5). Upon analyzing the confusion matrix, The diagonal of the confusion matrix usually represents the number of correctly classified samples, and the value on the non diagonal represents the number of incorrectly classified samples. Combined with confusion matrix analysis, there are 6 sample prediction errors in TWS (tea leaf spot) category, 2 sample prediction errors in N (leprosy) category, and the number of prediction errors in other categories is less than 1. The model exhibited remarkable accuracy in disease recognition tasks, achieving a specific value of 0.982301. Furthermore, the model attained an average precision rate of 0.964888, an average recall rate of 0.984068, and an average F1 score of 0.972617. Based on these experimental findings, we confidently conclude that this approach demonstrates excellent performance on existing datasets and holds significant potential for effectively classifying crop diseases and insect pests in practical farmland environments.

**Table 5:** Prediction results for the validation dataset

Class	Precision	Recall	F1 Score	Accuracy
EPM	0.966667	0.935484	0.950820	
HL	1.000000	1.000000	1.000000	
PM	1.000000	1.000000	1.000000	
SB	1.000000	1.000000	1.000000	
TSB	1.000000	0.990099	0.995025	
TALS	1.000000	1.000000	1.000000	
TWS	0.760000	1.000000	0.863636	
TWSB	0.988889	0.936842	0.962162	
TRRSD	1.000000	0.978261	0.989011	
N	0.933333	1.000000	0.965517	
Average	0.964888	0.984068	0.972617	0.982301

## Discussion

### Comparative analysis of the model in this study with current state-of-the-art image recognition methods

YOLOv9, published on February 21, 2024, is the most advanced method in the current image recognition field, and is a target detection system that uses programmable gradient information (PGI) learning [50]. PGI includes main branch, auxiliary reversible branch and multi-level auxiliary information. The auxiliary reversible branch is used to generate reliable gradients and update network parameters to solve the information bottleneck problem in deep learning. Multi level auxiliary information is used to aggregate gradient information of different prediction branches to alleviate the problem of information loss in depth supervision. In addition, YOLOv9 also uses the GELAN architecture, which combines the design of CSPNet and ELAN to achieve the balance of lightweight, reasoning speed and accuracy.

**Fig. 11:**
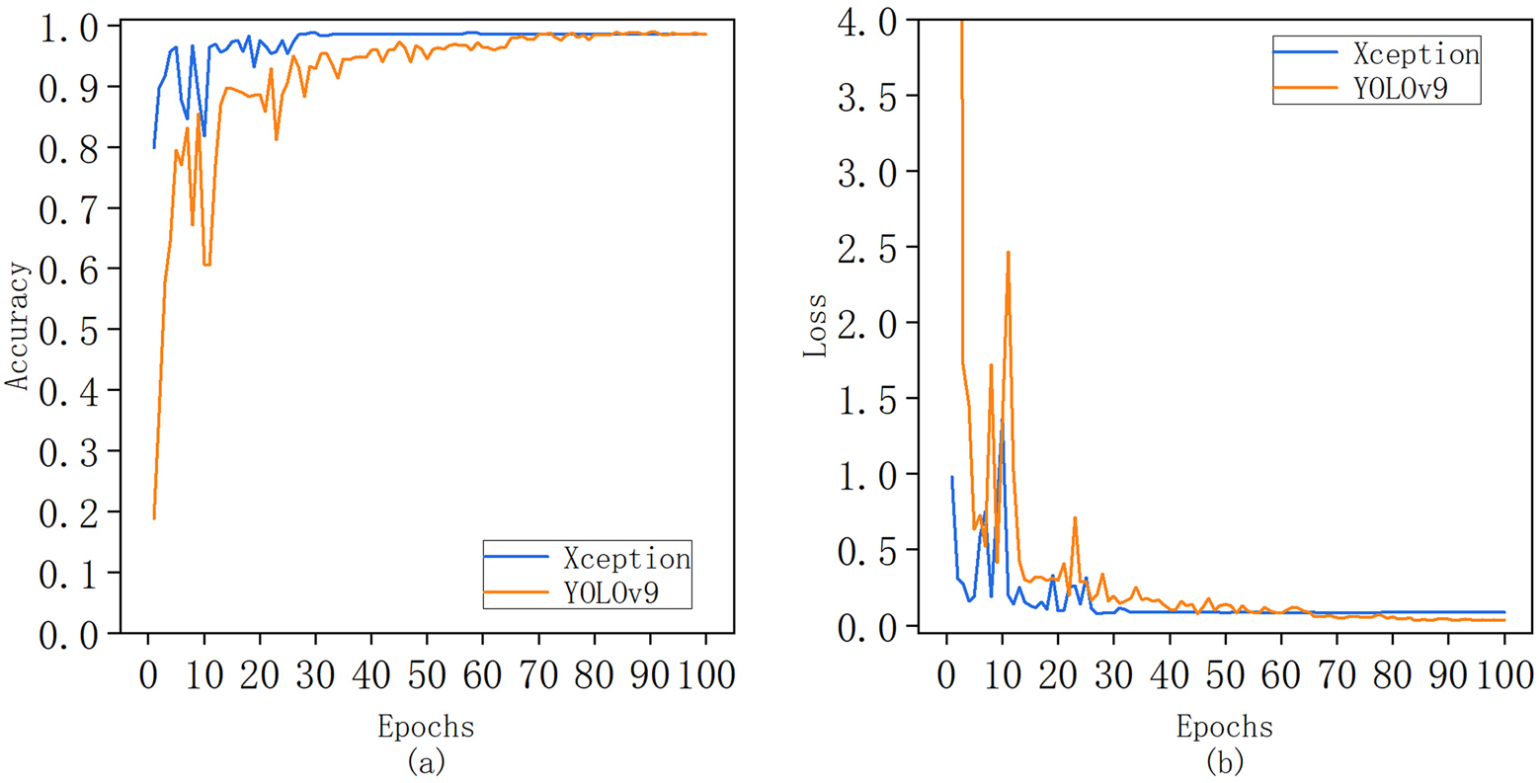
Curve of Accuracy and Loss Value of Xception Model and YOLOv9 Model after Fine Tuning Optimization(**a**) Curve of change in accuracy of test set; (**b**) Curve of change in loss function value of test set

Use YOLOv9 model and set the same super parameters as the finely optimized Xoption model to conduct classification training for diseases and pests of large leaf tea plants. The comparison of accuracy and loss function values is shown in Fig. 11 Curve of Accuracy and Loss Value of Xception Model and YOLOv9 Model after Fine Tuning Optimization(a) Curve of change in accuracy of test set; (b) Curve of change in loss function value of test set. It can be seen from the figure that the accuracy of the two models after convergence is the same, and the loss function value after YOLOv9 convergence is lower; The slightly optimized Xception model began to converge after 30 iterations, while YOLOv9 began to converge after 70 iterations and had severe shocks in the early stage of convergence.

Table 6 compares the relative performance of the fine tuned and optimized Xeption model with the current most advanced methods, and gives the evaluation indicators of the model, including the accuracy after convergence, the average loss after convergence, the time of each round, depth, total parameters and GFLOPs. The average precision of the two models after convergence is the same as 98.58%, and YOLOv9 is about twice as low as Xception in terms of the loss function value. Although YOLOv9 has fewer parameters, it is more than twice as high as Xcepion in terms of time, depth and GFLOPs in each round, and the model training efficiency is low.

**Table 6:** Comparison of performance between Xeption model and YOLOv9 model after fine tuning and optimization

Model	Average precision (%)	Average loss	Time of each epoch (s)	Depth	Total Parameters	GFLOPs
Xception	98.58	0.0849	27	81	20,881,970	16.7
YOLOv9	98.58	0.0440	90	291	8,999,114	42.8

To sum up, the optimized Xception model in this study has excellent performance on the large leaf tea plant disease and pest data set. Compared with the latest YOLOv9 network model, it has a high recognition accuracy and takes into account the lightweight design, and has successfully completed the task of disease and pest identification of agricultural microenvironment species.

### The novelty of this study

1. At present, no researcher has conducted in-depth research on diseases and pests of a specific tea variety. This work has conducted in-depth research on the identification of diseases and pests of large leaf tea plants as the raw material of Yunnan Pu'er tea, and the accuracy rate is higher than 98%, which is helpful for the intelligent, rapid and accurate identification of diseases and pests of tea, a special economic crop in Yunnan. At the same time, the previous research on tea disease recognition focused on diseases. This work studied four kinds of tea pests and collected images for recognition research.
2. Aiming at the problems of limited data scale, complex environment and difficult data collection in agricultural microenvironment, the efficiency of CNN development was significantly improved by adopting specific migration learning model architecture and frozen core training methods. At present, there are many advanced researches in the field of crop pest identification, such as image acquisition using high-end equipment such as hyperspectral images, multispectral images, UAV images, and model building using advanced algorithms such as YOLOv9. However, the cost of high-end image acquisition equipment is very high, and advanced algorithms have very high requirements for computer computing power, which cannot be applied in real-time in agricultural small environments. In this study, 9 kinds of diseases and pests of tea were identified at a very low cost. At the same time, the accuracy of advanced algorithms was not lost, which could be applied to real-time identification and classification of diseases and pests in tea gardens at a low cost.

## Limitations

1. There are many kinds of tea pests and diseases, and different kinds of pests and diseases have great differences in shape, color, texture, etc. This study only solved the problem of identification of pests and diseases in complex environment, but the performance of pests and diseases in terms of climate, soil, light and other factors of tea growing environment has not been studied. In the future, we will further collect more tea pictures under complex backgrounds such as different time periods, different illuminances and shades, enrich the data set of large leaf tea plant diseases and pests, and expand the model's ability to identify more types of diseases and pests.
2. This study is suitable for recognition tasks with small amount of data; On the contrary, if the data volume is large, training from 0 can effectively extract the characteristics of self built dataset. After collecting more images in the future, try to train the model from 0.

## Conclusion

Given the intricate characteristics of tea leaves, including their texture, shape, and color, as well as the numerous environmental interferences, accurate detection of diseases and pests poses a significant challenge. To address these complexities, this study employed a comprehensive approach that integrates deep transfer learning methods. Initially, data augmentation techniques were utilized to curate a comprehensive dataset specifically tailored for diseases and pests affecting Yunnan Big leaf kind of tea. Subsequently, leveraging existing convolutional neural network models and transfer learning principles, we developed a digital image-based automatic classification model specifically designed for the identification of diseases and pests in Yunnan Big leaf kind of tea, even under challenging environmental conditions. This model was further optimized through transfer learning techniques and the integration of an attention mechanism. The key findings of this study are summarized as follows:

1. Employing the transfer learning technique of "low-level freezing and high-level training" significantly enhances the model's accuracy. Through meticulous experimentation with freezing different layers, we discovered that unfreezing 16 layers and adopting a dynamic scheduling strategy for the learning rate yields optimal results. Specifically, the Xception model exhibited the highest accuracy, achieving a training accuracy rate of 98.58%, a recognition accuracy rate of 0.982301, an average precision rate of 0.964888, an average recall rate of 0.984068, and an average F1 score of 0.972617. These results demonstrate the effectiveness of our approach in accurately classifying diseases and pests in Yunnan Big leaf kind of tea, even under challenging environmental conditions.
2. When faced with the challenge of identifying diseases and pests in complex environments, the integration of an attention mechanism does not always guarantee an improvement in recognition accuracy. This can be attributed to several factors. Firstly, if the model has already reached a satisfactory level of fitting before introducing the attention mechanism, the addition of extra parameters may exacerbate the overfitting problem, ultimately reducing the model's performance. Secondly, in cases where the feature map size is small or the convolution operation is inappropriate, the attention mechanism may inadvertently introduce a significant amount of non-essential information, thereby hindering accurate recognition. Lastly, if the attention mechanism is positioned too closely to the classification level, it may become overly sensitive to variations in feature extraction, potentially compromising the classification decisions and leading to a decrease in recognition accuracy. Therefore, when incorporating an attention mechanism, it is crucial to carefully consider these factors to ensure its effectiveness in complex environmental conditions.
3. Addressing the challenges posed by limited data scale, intricate environmental conditions, and the difficulties associated with data collection in agricultural microenvironments, the utilization of specific basic model architectures and frozen core training methods can significantly enhance the efficiency of CNN development. When confronted with relatively small and unevenly distributed training datasets, the key to achieving high model accuracy lies in the employment of data augmentation techniques and transfer learning strategies. By leveraging these techniques, we can overcome the limitations of limited data and complex environments, enabling us to develop robust and accurate CNN models for agricultural applications.

In this study, we successfully enhanced the model's accuracy and F1 scores by meticulously adjusting training super parameters and integrating the ImageNet dataset with our unique training parameters. However, it's crucial to acknowledge that our test datasets are somewhat limited in size and exhibit uneven sample distribution, which, to a certain extent, constrains the comprehensiveness and generalizability of our findings. The datasets utilized in this paper was primarily collected during early morning hours under sunny weather conditions. As we move forward, our research will focus on gathering a more diverse set of tea images, encompassing various time periods, light intensities, shading, and other complex backgrounds. This will enrich our Big leaf kind of tea dataset, broaden the model's capability to identify a wider range of diseases and pests, and potentially facilitate its deployment on mobile devices. By doing so, we aim to enhance the model's popularity and application, addressing critical disease prevention and control issues, and ultimately providing robust support for the sustainable development of the tea industry.

### Supplementary Information

Supplementary material 1

Supplementary material 2

Supplementary material 3

## Data Availability

The datasets used in the current study are available to corresponding authors upon reasonable request.
